# Phase 3 LILAC study sets standard for clinical evaluation of oncology biosimilars

**DOI:** 10.18632/oncotarget.26528

**Published:** 2019-01-01

**Authors:** Hans-Christian Kolberg, Gunter von Minckwitz, Vladimir Hanes

**Affiliations:** Hans-Christian Kolberg: Marienhospital Bottrop gGmbH, Bottrop, Germany

**Keywords:** biosimilars, breast cancer, trastuzumab, ABP 980

ABP 980 (Amgen Inc, Thousand Oaks, CA, USA) is a biosimilar of trastuzumab, the recombinant humanized monoclonal antibody against human epidermal growth factor receptor-2 (HER-2), which is overexpressed in 15-30% of patients with breast cancer [1, 2] and 10-30% of patients with gastric tumors [2]. ABP 980 (KANJINTI™, Amgen Europe B.V., The Netherlands) was approved by the European Medicines Agency in March 2018 for the same indications as the originator biologic or reference product (RP), Herceptin^®^ (trastuzumab; Roche Registration GmbH, Germany), including treatment of HER2-positive (HER2+) metastatic breast cancer, HER2+ early breast cancer and HER2+ metastatic adenocarcinoma of the stomach or gastroesophageal junction [3, 4].

In accordance with the regulatory requirement of a totality-of-evidence (TOE) approach to gaining approval of biosimilarity [5, 6], a body of evidence now exists to support similarity of ABP 980 with its reference product (RP) trastuzumab. Structural, functional, and pharmacologic assessments have demonstrated a high degree of similarity between ABP 980 and trastuzumab RP including comparable binding to the extracellular domain of HER2 and blocking of receptor activation [7]. In addition, pharmacokinetic studies have demonstrated a similar PK profile without any difference in immunogenicity or safety [8]. Most recently, the phase 3 LILAC study provided the first clinical safety and efficacy data of ABP 980 versus trastuzumab in women with HER2-positive early breast cancer, adding to the totality of evidence for this biosimilar.

The LILAC study was a large, double-blind, randomized, multi-national, multi-center trial in women with HER2+ early breast cancer. The study included women with histologically confirmed HER2-positive invasive early breast cancer with tumors of at least 2 cm. After a 28-day screening period, patients received 12 weeks of run-in chemotherapy with epirubicin and cyclophosphamide, following which they were randomized to receive ABP 980 or trastuzumab as HER2-directed therapy. The co-primary efficacy endpoints in this study were risk difference and risk ratio (RR) of pathologic complete response (pCR) in breast tissue and axillary lymph nodes, for the primary analysis assessed at a local laboratory. In addition to local laboratory evaluation of tumor samples, a sensitivity analysis was conducted based on central pathology findings to reduce variability between local pathologists. LILAC is the only multicenter multinational neoadjuvant breast cancer study to include evaluation of tumor samples at a central laboratory. Secondary endpoints included risk differences and RRs for pCR in breast tissue only and risk differences and RRs for pCR in breast tissue and axillary lymph nodes in the absence of ductal carcinoma in situ. Safety assessments included incidence of treatment-emergent adverse events, cardiac safety assessments, including LVEF and immunogenicity response [9].

The primary efficacy endpoint results of the LILAC study showed pCR in 172 (48%) of 358 patients who received ABP 980 and 137 (41%) of 338 who received trastuzumab, yielding a risk difference of 7.3% (90% CI 1.2-13.4) and relative risk (RR) of 1.188 (1.033-1.366). When assessed locally, the upper bounds of both 90% CIs slightly exceeded the predefined equivalence margins of 13% and 1.318, respectively. In the central laboratory sensitivity assessment, pCR was seen in 162 (48%) of 339 and 138 (42%) of 330 patients in the ABP 980 and trastuzumab groups, respectively. The results based on central review were within the pre-set margins (risk difference 5.8%, 90% CI -0.5 to 12.0, and RR 1.142, 0.993 to 1.312).

The frequency, type, and severity of AEs were comparable between treatment arms and were consistent with the known safety profile of trastuzumab. Cardiac safety is of particular concern with trastuzumab. Overall, seven patients had cardiac failure as adverse events during the neoadjuvant phase (six patients in the ABP 980 group and one in the trastuzumab group), and none of the cardiac failure events were coincident with decreased LVEF. All cardiac failure events were grade 1 or 2, and patients were able to complete all planned doses of ABP 980 or trastuzumab with no worsening of symptoms. Importantly, no patients discontinued due to cardiac failure in the adjuvant phase, suggesting that the potential for cardiotoxicity is low and no higher than that reported with trastuzumab RP. Immunogenicity analysis showed that formation of antibodies was low and similar for both treatment arms [9].

Several important features of the LILAC study set it apart from other studies of trastuzumab biosimilars. First, the LILAC study is the only study in breast cancer to incorporate a sensitivity analysis regarding pCR that was based on central independent evaluation of tumor samples (as opposed to local review only). Although central review of samples is considered to be logistically challenging, it has the potential to reduce inter-pathologist variability, particularly in a multicenter, multinational study [9].

Secondly, the LILAC study is the only study to include both a neoadjuvant and adjuvant phase and explore the potential clinical effects of switching from trastuzumab reference product to ABP 980. This approach mimics real-world clinical use of biosimilars, in which patients may be switched from trastuzumab reference product to ABP 980. In the LILAC study, half the patients treated with trastuzumab during the neoadjuvant phase remained on trastuzumab in the adjuvant phase, while the other half were switched from trastuzumab reference product to ABP 980. The safety, tolerability, and immunogenicity risks associated with switching was found to be low, with only 8% of patients who switched to ABP 980 treatment having grade 3 or worse adverse events and only 2% testing positive for new binding antibodies. These safety and immunogenicity results were similar to those in patients who continued on trastuzumab (without switching) [9].

The totality of evidence for ABP 980 supports the scientific rationale of extrapolation across all trastuzumab indications. Extrapolation bridges the data collected from one indication for the biosimilar product to all approved indications for the originator biologic, if the mechanism of action (MOA) is the same. Trastuzumab is approved for use in breast cancer as well as HER2+ gastric carcinomas based on its common MOA mediated via HER2 across these cancers. The totality of evidence supports the similarity of ABP 980 and trastuzumab including a similar MOA, thus providing scientific justification for extrapolation [7-10].

Increasing our understanding of biosimilars will lead to a higher level of comfort with their use in the clinic. As more biologics lose patent protection, we may expect an increase in the availability and use of biosimilars and, as they become an important treatment option, education regarding their use will be a key factor in assuring safe and potentially cost-effective treatments for our patients.

## References

[1] Burstein HJ (2005). New England Journal of Medicine.

[2] Iqbal N (2014). Mol Biol Int.

[3] KANJINTI™ Summary or Product Characteristics.https://www.medicines.org.uk/emc/product/9233/smpc. Accessed October 31, 2018

[4] Herceptin^®^ (trastuzumab). Summary of Product Characteristics.https://www.ema.europa.eu/documents/product-information/herceptin-epar-product-information_en.pdf. Accessed October 31, 2018

[5] European Medicines Agency (2015). Guideline on similar biological medicinal products containing biotechnology-derived proteins as active substance: non-clinical and clinical issues.

[6] Christi L FDA’s Overview of the Regulatory Guidance for the Development and Approval of Biosimilar Products in the US.

[7] Hutterer K

[8] Hanes V (2017). Cancer Chemother Pharmacol.

[9] von Minckwitz G (2018). Lancet Oncology.

[10] Kolberg HC (2018). Eur J Cancer.

